# Diffusion of flue gas desulfurization reveals barriers and opportunities for carbon capture and storage

**DOI:** 10.1038/s41467-020-18107-2

**Published:** 2020-08-27

**Authors:** Stijn van Ewijk, Will McDowall

**Affiliations:** 1 Center for Industrial Ecology, School of the Environment, Yale University, New Haven, CT 06511 USA; 2 Institute for Sustainable Resources, University College London, London, WC1H 0NN UK; 3 Department of Civil, Environmental & Geomatic Engineering, University College London, London, WC1E 6BT UK; 4 Energy Institute, University College London, London, WC1H 0NN UK

**Keywords:** Climate-change mitigation, Power stations

## Abstract

Addressing climate change may require rapid global diffusion of Carbon Capture and Storage (CCS). To understand its potential diffusion, we analysed a historical analogy: Flue Gas Desulfurization (FGD) in the global coal power market. Our findings challenge common patterns: diffusion of FGD is not described by a single S-curve but by multiple steps and does not slow down after materiality. The regulation-driven diffusion of FGD can be fast, especially for retrofit since it does not require new power plants. Owing to the mature size of coal power plants, the diffusion of FGD is driven by unit numbers instead of unit capacity growth. We find that the diffusion of CCS in climate change mitigation pathways, when normalised for economic growth, rarely exceeds the historical maximum diffusion rate of FGD. Our findings suggest that end-of-pipe abatement technology can diffuse fast and to a great extent provided deep, consistent long-term regulatory commitment.

## Abstract (web summary)

The historical diffusion of SO_2_ control technology gives insights into the potential uptake of carbon capture and storage. Here the authors show that the global diffusion of flue gas desulfurization technology was very fast at times, especially for retrofit, and even after materiality, but strongly depended on regulation.

## Introduction

The Paris Agreement aims for limiting warming to 1.5 °C^1^, which is a geophysical possibility but requires historically unseen rates of emission reduction, and a lack of early action may necessitate a consecutive reduction effort that is technically impossible^2^. Various pathways for achieving the target rely on extensive use of CCS^3^; an understanding of the diffusion of this technology is therefore critical. Already, there is a growing literature on the rates of diffusion of energy and climate change abatement technologies^4–8^.

The case of Flue Gas Desulfurization (FGD) provides a relevant historical analogy for end-of-pipe technologies such as Carbon Capture and Storage (CCS). Both technologies aim to remove harmful emissions from flue gases (sulfur dioxide (SO_2_) and carbon dioxide (CO_2_)), to then be stored, used as a by-product, or disposed of (in the case of FGD). Despite clear differences (notably the need for carbon dioxide transportation and storage infrastructure), FGD and CCS have important similarities regarding economic and financial viability, politics, policy and regulation^9^, and costs and scaling dynamics^10^. Our analysis of FGD therefore provides insights into the plausible diffusion patterns for CCS.

Speed and scaling are among the major uncertainties regarding the viability of CCS^11^. More immediately, the global diffusion of FGD and its implications for CCS can inform and improve integrated assessment models (IAMs), since many IAM scenarios for meeting global climate targets rely heavily on the application of CCS. The validity of the assumed rates of diffusion of CCS is critical to the credibility of such scenarios. In literature comparisons of historical and modelled energy system change, CCS is the only major technology that lacks historical data on capacity additions—a historical analogy such as FGD is therefore useful.

The literature on the diffusion of end-of-pipe abatement technology focuses on the role of costs, regulatory stringency, firm heterogeneity and regulatory design in facilitating rapid diffusion^12–14^. Such studies typically treat policy as a static instrument (whether market-based or command-and-control), rather than an evolving process; besides, many are specific to a single country or small group of countries. Previous work on sulfur control studied country-level development of sulfur control technologies in various jurisdictions^7,10,11,15–17^. These studies rely on data from a small number of countries and few make the important distinction between retrofit and new build FGD.

Our study takes a longer-term and geographically broader perspective and contributes to the literature on global long-term patterns of energy technology diffusion^4,6,18–21^. We focus on end-of-pipe abatement technology and consider the importance of market growth^5^ by analysing diffusion both in terms of absolute capacity and market share. We also consider competing technologies, which are frequently overlooked^22^; for FGD, the main competitors are Fluidised Bed Combustion (FBC) and an Integrated Gasification Combined Cycle (IGCC), both of which can reduce SO_2_ emissions without flue gas cleaning^23^. Although the reasons for pursuing FBC and IGCC are more diverse than SO_2_ emission reduction, the technologies do affect demand for FGD and therefore need consideration.

In this article, we show that various patterns commonly found for energy conversion technologies also hold for FGD; at the same time, we challenge assumptions regarding the incremental nature of diffusion and Kramer and Haigh’s idea that diffusion slows when energy technologies reach materiality^24^. Our global analysis is based mainly on a coal power database^25^ that covers capacities and (de-)commissioning dates of coal power units and the year of introduction of FGD. We measure the diffusion of FGD from 1970 to 2010 and consider the contributions of unit numbers, unit size, type of FGD (retrofit versus new build) and competing technologies (FBC and IGCC); we also study the spatial and temporal patterns of diffusion and compare the historical diffusion of FGD with modelled diffusion of CCS. Based on our findings, we formulate implications for energy modelling and policymaking.

## Results

### Diffusion of sulfur control is stepwise

**Fig. 1:**
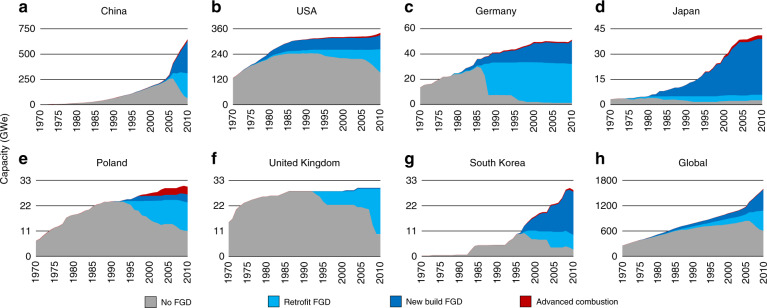
Diffusion of sulfur control in the coal power sector of selected countries and globally. **a**–**g** show capacities for the largest national coal power sectors with significant uptake of FGD. **h** shows global coal power capacities. Electric capacity (GWe) of coal power units without FGD (grey), with retrofit FGD (light blue), new build FGD (dark blue), or advanced combustion technology (red). The scales for the vertical axes vary by panel. Source data are provided as a Source Data file.

We first analysed trends in total sulfur control in the biggest national coal power markets, excluding those with large amounts of coal power but hardly any FGD (India and Russia). Figure 1 shows the diffusion of coal capacity types for seven countries and globally, by presence and type of sulfur control. The countries cover 72% of global coal power capacity in 2010. There is a distinction between units without FGD (no FGD), those retrofitted with FGD (retrofit FGD), new builds with FGD (new build FGD) and FBC/IGCC (advanced combustion) units. The procedure for allocating coal power units to types of sulfur control is described in ‘Methods’.

The charts in Fig. 1 suggest stepwise adoption of sulfur control technologies, linked directly to major legislative steps, which confirms the regulation-driven nature of pollution control technologies^15^. In the United States, a series of legislative initiatives put in place strong regulatory drivers for sulfur abatement. The first major piece of legislation was the 1963 Clean Air Act, followed up by the 1967 Air Quality Control Act and the 1970 Clean Air Act Amendments. The 1977 Clean Air Act Amendments effectively required FGD and led to the rapid installation of FGD in US power plants.

The chart for Germany reveals a rapid increase in retrofit FGD in the 1980s, explained by comprehensive regulations introduced in 1983 in response to forest dieback^26,27^. The European Union, building on the German experience, introduced the Large Combustion Plant Directive (LCPD) in 1988^28^, which required the installation of FGD at many power plants, as evidenced by the trends in the 1990s for Germany, Poland and the UK. The UK chart, moreover, reveals a rapid increase in FGD to meet the 2008 deadline of the 2001 update to the LCPD.

In Japan, FGD was first adopted in the 1970s and rapidly rolled out in the 80s and 90s, including for facilities other than power plants. This development was boosted by the oil crisis, which led to high prices for low-sulfur oil and therefore made FGD a relatively affordable abatement option. The technology was largely imported from Germany and the US but domestic technological improvements turned Japan into an exporter^29^. Neighbouring South Korea followed a similar trajectory. Finally, China started adopting FGD at a large scale only recently, with 2004 legislation requiring FGD on all new build coal power plants^30^.

The charts suggest that once FGD was introduced it became the standard for new plants. In countries where total coal power capacity grew quickly (South Korea, Japan), retrofit grew only gradually, possibly reflecting the higher costs of retrofit FGD, whether measured as investment cost^31,32^ or levelized cost per tonne of SO_2_ removed^32^. In countries where total coal power capacity grew slowly (Germany, Poland, UK, US), FGD also became the norm for new capacity, but retrofit had to play a much greater role in achieving universal sulfur control. For China, the distinction between new build and retrofit should be interpreted carefully because many new builds were retrofitted very shortly after commissioning.

### FGD evades materiality and partially the S-curve

**Fig. 2:**
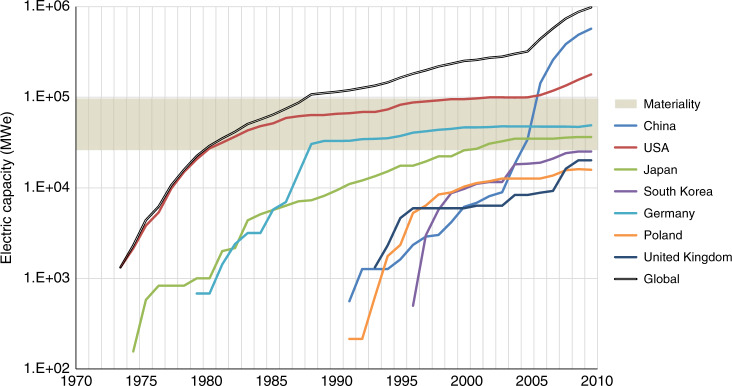
Installed electric capacity of coal power with FGD in selected countries and globally. The horizontal bar indicates materiality, defined as 1% of global primary energy consumption. The scale on the vertical axis is logarithmic because the contribution of different countries varies by an order of magnitude. Source data are provided as a Source Data file.

The second step of our analysis focused on the rate of diffusion of FGD. Figure 2 shows the diffusion of FGD (on a logarithmic scale) for the selected countries and globally. Kramer and Haigh^24^ argued that energy technologies obey a law that limits the growth rate once a technology has reached materiality, defined as 1% of global primary energy use. Figure 2 shows the electric capacity of coal power equivalent to materiality as a horizontal bar. Since primary energy use grows over time, we calculated a range (see ‘Methods’). We show that global diffusion indeed slows down, but accelerates again in the mid-2000s, breaking the law posited by Kramer and Haigh, as a result of growing stringency of sulfur regulation, mostly in the US and China.

Diffusion of innovation is generally found to follow an S-shaped curve that is best approximated by a three-parameter logistic curve^20^; such curves have been successfully applied to a range of energy and pollution control technologies^10,20,33,34^. Logistic growth involves near-exponential growth in the early stages of diffusion and a subsequent slowing of the growth rate as the market approaches saturation. We ran regressions of logistic curves and calculated indicators for the rate and extent of diffusion (see ‘Methods’). The rate of diffusion was expressed by calculating delta *t* (*∆t*), which is the number of years between 10 and 90% of the saturation level. The extent of diffusion was expressed by the saturation level of the logistic curve, either in terms of absolute capacity or share of the total market. Supplementary Table S1 summarises the regression results.

We found that the rate of diffusion is very different for different countries and somewhat different depending on whether measuring installed capacity or market shares. At the global level, ∆*t* is 26 years for capacity and 44 years for market share (*R*^2^ = 0.93). The figure for shares is higher because it is partly a function of new capacity without FGD, i.e., the addition of capacity without FGD lowers the share of FGD coal capacity, all else being equal. For the selected countries, which are leaders in terms of FGD, ∆*t* is 5–25 years for capacity and 4–16 years for shares (including only regressions with *R*^2^ > 0.95). For a single country, ∆*t* is similar for shares and capacity, but diffusion times vary widely between countries; moreover, global diffusion is slower than national diffusion because it is affected by the lag in adoption between countries.

At the global level—and within several major countries—the diffusion of FGD diverges from the classic S-shaped curve and, as a result, has a lower fit with the logistic regression. We observed pulses of rapid diffusion, largely driven by stepwise increases in retrofit FGD. The resulting stepwise diffusion can be observed for the US, UK, Germany and South Korea. It is also apparent at the international level with later-regulating countries driving strong increases in the global capacity of diffusion. The stepwise nature of diffusion cannot be conclusively identified for smaller countries since markets with few power plants inherently exhibit staggered adoption. Supplementary Fig. S1 shows that two or three pulses can be observed for the US, UK, Germany and South Korea. Supplementary Table S2 shows that a logistic regression of a single pulse of diffusion for South Korea and Germany yields a much smaller ∆*t* than a regression over the entire period.

Explanations for the S-curve assume a population of adopters making independent adoption decisions^35,36^. When adoption decisions are strongly influenced by a single actor, such as a national government, the diffusion curve may deviate from the S-shape. For example, diffusion can be exponential when the required information for adoption is broadcasted instead of transferred from users to non-users because it enables simultaneous adoption anywhere in the population^37^. The observed pulses in the diffusion of FGD are consistent with the expectations associated with the number of decisionmakers: whereas the total population of decisionmakers in the coal power sector may be large, their decisions are heavily influenced by those of a single government.

The punctuated pattern of diffusion is also consistent with a pattern of policy change in which periods of policy stability are interspersed with substantial policy changes (as described in punctuated equilibrium theory^38,39^). In the case of FGD, a policy change leads to a burst of diffusion within a subset of the country’s population of coal power units. After a period of stability, a second policy change drives a subsequent burst of diffusion. Such spatial heterogeneity in adoption patterns is likely when regulation plays a central role in driving diffusion, which is common for end-of-pipe pollution control^7,13–15^. In cases where adoption is smooth (e.g., Japan), the emphasis is on new build, which is subject to sulfur regulation but also strongly shaped by market decisions regarding the construction of new coal power.

### Retrofit can diffuse particularly fast

In the third step, we looked at explanations for fast instances of diffusion. The stepwise pattern of diffusion suggests FGD can diffuse very quickly when demanded by government regulations. Based on the observed maximum adoption rates, it appears to be technically possible to fit a national coal power fleet with FGD within a few years only (Germany increased its share of FGD from 10 to 79% in 4 years). In particular, for late adopters, the formative phase^4^ of experimentation may be absent, and adoption can be fast from the start. For example, South Korea features immediate and rapid growth, benefiting from technology spill-overs arising from experience in Germany, the US and neighbouring Japan.

For new build FGD, which requires new power plants, growth rates are constrained by coal power market growth. Despite being technically less practical, retrofit FGD is therefore able to diffuse more rapidly, as evidenced by the rapid pulses of retrofit FGD diffusion in Fig. 1, most prominently in Germany and the UK. At the global level, 5-year average growth factors for installed capacity from 1980 to 2010 tend to be higher for retrofit than for new build (see Supplementary Fig. S2). At the country level, logistic regressions of installed capacity of retrofit and new build FGD feature an *R*^2^ of over 0.95 for only three countries, but in all cases, the ∆*t* for retrofit is shorter than for new build (see Supplementary Table S3).

The high rates of diffusion can be partly explained by the lack of alternatives for sulfur control besides FGD. The regulatory changes that spurred diffusion of FGD often either directly required end-of-pipe abatement or mandated emission reductions that, at the time, could only be feasibly achieved through FGD. For the US, it was previously found that the strict regulations equated ‘picking a technology winner’ and that private investment and technology learning would not have occurred without it^40^. This is likely to be the case for all countries in our sample since diffusion closely follows the introduction of regulatory requirements. The alternative abatement options of IGCC and FBC play a limited role (Poland, with 11%, had the highest share of FBC in 2010); however, coal washing and fuel switching as an alternative to FGD cannot be ruled out based on our data (see ‘Methods’).

### FGD unit scaling has limited impact

**Fig. 3:**
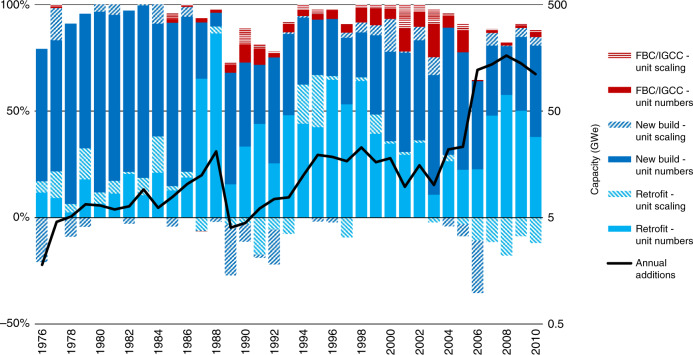
Contributions to annual additions of global coal capacity with sulfur control. The bar chart shows the relative contribution of number, size and type of coal power units to annual additions of global coal power capacity (primary axis). The line chart shows the annual additions (GWe) on a logarithmic scale (secondary axis). Source data are provided as a Source Data file.

In the fourth step, we looked at contributions to capacity additions. We do not show explanatory variables but reveal how sulfur control increased annually through an increase in unit numbers or unit size of new build FGD, retrofit FGD, or advanced combustion (FBC/IGCC). Figure 3 shows the relative contributions of unit numbers, size and type (the stacked bars) to annual global capacity additions (the black line) of sulfur control in 1976–2010, based on decomposition analysis (see ‘Methods’). Negative values occur when added capacity has a smaller average unit size than preceding annual additions; the drop after 2005 is caused by relatively small Chinese units. The sum of the absolute values of the contributions explains 100% of the annual capacity growth.

Unit scaling of technology is part of the natural development of many technologies^41^ and typically takes place after a dominant design^42^ has been established^43^. A typical pattern is that unit numbers drive diffusion in the formative phase, with unit scaling becoming prevalent in a subsequent up-scaling phase, after which unit numbers become the main driver again in the growth phase^4^. For the diffusion of FGD, these phases are linked to the same phases in coal power technology development, because both retrofit and new build FGD units inherit their capacity from coal power units. FGD started in the early 70s when coal power entered the growth phase^4^ and most unit scaling had therefore already happened.

As a result of the maturity of coal power technology, unit numbers consistently contributed more than unit scaling to growth in total sulfur control capacity. In any given year, unit scaling contributed at most 35% to growth in total sulfur control. For retrofit, unit scaling was more prevalent in the early years, reflecting the maturation of the size of coal power units and the transition from the up-scaling phase to the growth phase. Unit scaling has a relatively large contribution for retrofit (compared to new build) because coal power plants that are retrofitted are older and therefore more likely to date from the up-scaling phase of coal power. Because of modular design, individual components of FGD systems may have scaled more slowly than overall FGD systems.

The average unit size of installed capacity of new build FGD was 1.8 (1.2–3.5) times larger than retrofit FGD. This is because coal power units have grown over time and retrofit FGD inherits unit size from coal capacity constructed, on average, decades earlier. Due to its relatively large unit size, new build FGD units on average contribute more than retrofit to the overall share of FGD. However, new build FGD cannot be concluded to be preferable because it raises absolute emissions of SO_2_ by increasing total coal power capacity (unless replacing retired coal power capacity). This paradoxical effect—an increase instead of a decrease of emissions upon adopting new builds with pollution control technology—should be considered for any end-of-pipe technology.

### The spatial and temporal diffusion of FGD

In the fifth step, we analysed the validity of established insights regarding duration, extent and time of adoption of energy technologies, as described in various studies^6,18,34^ including one regarding FGD in the US, Japan and Germany^10^. We ran regressions for the largest coal-consuming countries in the database but excluded countries that had no FGD as of 2005. We also excluded regressions for time series reaching <60% of the saturation level (extent) or with an *R*^2^ lower than 0.95, consistent with previous literature^10,34^. The resulting sample size is 23 countries.

**Fig. 4:**
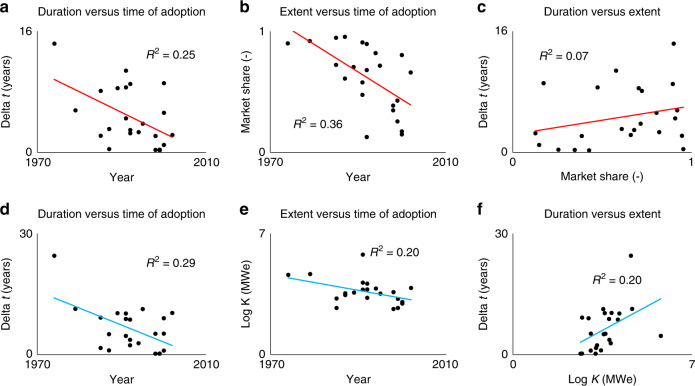
The relationships between duration, extent and time of adoption of FGD. **a**–**c** show the relationships for the diffusion of FGD measured in terms of market share. **d**–**f** show the relationships for the diffusion of FGD measured in absolute capacity. Source data are provided as a Source Data file.

The duration and extent of diffusion were measured by respectively the ∆*t* and the saturation level of the logistic regression; the time of adoption was defined as the first recording of a coal power unit with FGD (which may have been preceded by pilot and demonstration facilities). Figure 4 summarises the correlations between the three variables for both market share (**a–c**) and absolute capacity (**d**–**f**) for coal power with FGD (retrofit and new build). For capacity, the extent was plotted as the log of capacity (MWe) (**e**, **f**).

Consistent with the literature, we find that later adopters are faster adopters (**a**, **d**), that the extent is smaller for later adopters (**b**, **e**), and that diffusion to a greater extent takes longer (**c**, **f**). The difference in correlations between market share and capacity metrics is partly caused by underlying trends in market size, which affect the two indicators differently. Specifically, late adopters tend to have smaller coal power markets (Supplementary Fig. S3), which makes the limited extent of diffusion for later adopters more prominent when measuring absolute capacity, and partly explains why late adopters can adopt faster (other explanations include, among others, the relative maturity of the technology upon diffusion).

According to the literature, the spatial diffusion of technologies tends to be slow but pervasive in core regions, followed by faster but less pervasive diffusion in rim and periphery regions^4,20,44^. To identify such trends for the diffusion of FGD, we define regions by year of introduction of FGD: core (1970s), rim (1980s) and periphery (1990s and 2000s). Surprisingly, the core region consists of countries that are far apart: the US, Germany and Japan. However, they were also the industrial powerhouses of the day, and spill-overs have likely occurred. Less surprisingly, the rim region consists of mostly European states that border with Germany. The periphery includes European countries further from Germany, but also many other countries all over the globe—at this point, transfers and spill-overs could occur in many directions.

### CCS modelling is mostly consistent with FGD experience

Finally, in the sixth step, we assessed whether the rates of diffusion of CCS in climate change mitigation models are consistent with the historical experience from FGD. Recent studies have compared the historical and modelled diffusion of various technologies in IAMs but, although some cover both FGD and CCS^7^, none provide a direct comparison^5,7,45,46^. Following previous work that suggests a larger economy enables faster diffusion because industrial capacity scales with the overall economy^5,46^, we conduct our analysis using both absolute (GW/decade) and normalised deployment rates (GW/decade/$T GDP).

We find, first of all, that FGD has diffused faster than any other observed power sector technology: the normalised historical global maximum (in GW/decade/$T GDP) is higher for FGD than for coal and oil, natural gas, nuclear, or any renewable energy technology (see Supplementary Table S4 for a detailed comparison). This implies that comparisons between CCS and energy technologies that are not FGD, as presently available in the literature^5^, lead to comparatively low limits to feasible CCS diffusion, all else being equal. Our comparison between CCS and FGD assumes generous limits to the diffusion of CCS because FGD has historically diffused faster than any other energy technology.

We compared historical FGD diffusion with modelled diffusion of CCS in 1.5 °C and 2 °C average global warming scenarios (see ‘Methods’). We used the scenario database^47^ from the IPCC’s Special Report on 1.5 °C of Global Warming (SR15)^3^, which provides data on coal, gas and bioenergy CCS (BECCS) deployments in these scenarios. This database provides suitable data for only three models. We therefore expanded the analysis by also examining CCS deployments in the 450 ppm scenario (approximately equivalent to a 2 °C degree scenario) from the AMPERE scenario database^48,49^. For many models, scenario data are only available in 10-year time steps, and our comparison therefore relies on decadal averages.

**Fig. 5:**
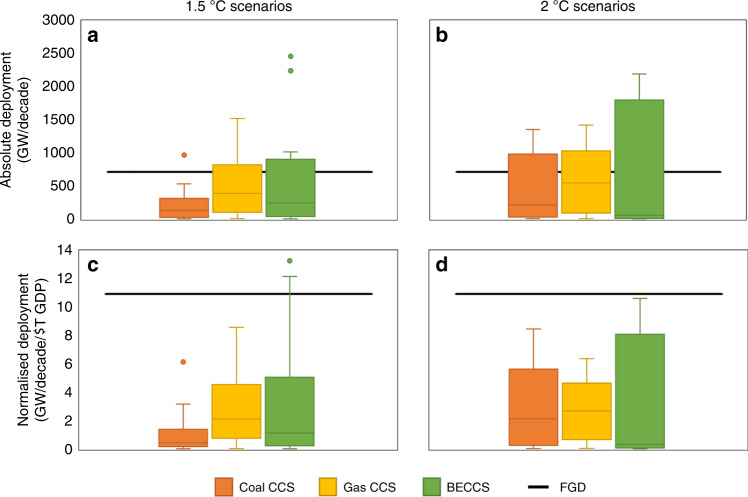
Comparison of modelled and historical maximum deployment of CCS. The comparison includes coal, gas and bioenergy CCS (BECCS). Deployment of CCS is measured in absolute capacity (**a**, **b**) and capacity normalised by income (**c**, **d**). The modelled data are for 1.5 °C (**a**, **c**) and 2 °C (**b**, **d**) climate change scenarios. Sample sizes for the 12 box plots in **a**–**d**, reading each panel left to right: *N* = 33, 33, 23, 37, 33, 27, 33, 33, 23, 37, 33, 27. Boxes show the median and interquartile ranges of scenario data. Whiskers show min/max values that lie within 1.5 times the interquartile range, and scenarios outside this range are plotted as outliers. An outlier is excluded from the 2 °C scenarios of coal CCS—the IMACLIM AMPERE 450 scenario reached >4000 GW/decade (absolute), and >36 GW/decade/$T GDP (normalised). The 1.5 °C scenarios are taken from the IMAGE, REMIND and AIM/CGE models; 2 °C scenarios include these, and also scenarios from DNE21, POLES, MESSAGE, MERGE-ETL, WITCH and IMACLIM. Source data are provided as a Source Data file.

Figure 5 shows the maximum global historical FGD diffusion rate (horizontal bar) and the diffusion rates of CCS in 1.5 °C and 2 °C scenarios (the box and whisker plots). Panels **a** and **b** reveal that absolute global rates of CCS deployment in many scenarios exceed the recent rapid global diffusion of FGD (731 GW/decade). However, when normalised to control for growth in the global economy, **c**, **d** show that most scenarios feature lower growth than the historical experience for FGD (11 GW/decade/$T GDP). The maximum deployment rate for each model is listed in Supplementary Tables S5–S7. Assuming CCS should be expected to diffuse no faster than FGD, our findings suggest that the modelled pathways of CCS deployment are valid.

Consistency between historical FGD diffusion and modelled CCS diffusion does not necessarily imply scenario feasibility. Whilst retrofit played a critical role in the rapid diffusion of FGD, many of the modelling scenarios for CCS fail to distinguish between retrofit and new build, which complicates the comparison. Moreover, whilst the analogy between FGD and CCS is useful, there are significant differences between them (whether applied to coal or gas), which should affect expected diffusion rates; this is reflected on in more detail in the discussion section. We have included BECCS given strong policy and research debate about the feasibility of rapid BECCS deployment in IAM scenarios, but our comparison should be treated with caution: an analysis of the unique constraints on power generation from biomass – including concerning land-use change and competition with food production – are outside of the scope of this study.

## Discussion

We analysed historical diffusion patterns of FGD technology in the global coal power sector and established key patterns that may be unique to end-of-pipe abatement technology and of great relevance to the future development of CCS. Some of our findings for FGD suggest CCS could diffuse rapidly and to a great extent. The regulation-driven nature of FGD often leads to stepwise diffusion with rapid pulses that suggest high possible rates when policies demand abatement. At the global scale, FGD diffusion does not obey Kramer and Haigh’s materiality law since growth increases instead of decreases after coal power with FGD represented ~1% of global primary energy.

Given strong regulatory policies, it seems plausible that CCS could diffuse at rates like those observed for FGD, leaving aside challenges of transport and storage uniquely faced by CCS. The observed stepwise diffusion of FGD aligns with literature findings that regulatory standards, rather than any other policy instruments, such as public R&D, are critical to the diffusion of FGD^13,15^. At the same time, strict regulatory standards can result in the retirement of polluting capacity^50^, and further analysis would be required to establish the extent to which FGD regulations had this effect too, and whether analogous patterns should be expected for CCS in the case of strict regulatory standards for carbon capture.

Whilst new build FGD is physically constrained by coal power market growth, retrofit is not, which has led to particularly rapid diffusion in some instances, though such rapid diffusion rarely went uninterrupted till full or near market saturation. Another feature of FGD and CCS, in comparison with the diffusion of other energy technologies, is that unit scaling contributes little to overall capacity growth since the technologies are constrained by the mature size of the host power plant. In the absence of coal power unit scaling, diffusion of CCS will depend almost solely on the number of installations.

The relatively fast diffusion of retrofit FGD is promising for CCS since the diffusion of retrofit CCS in the coal power sector helps to avoid the stranding of existing power generation assets that are otherwise incompatible with decarbonisation objectives. Moreover, constructing new coal power plants is not compatible with stringent climate targets, such as net-zero by 2050; CCS is not perfectly efficient (neither is FGD) and therefore cannot reduce plant emissions to zero. Besides, mining and transport of coal also cause emissions. The experience of the rapid adoption of retrofit FGD suggests that it is important to understand better the possible diffusion and role of retrofit CCS, and we believe integrated assessment models should more often explicitly model CCS retrofit.

Our findings can largely be explained by the broad techno-economic characteristics of FGD: retrofit is likely more costly than new build, the unit size is inherited from the host power plant, and diffusion is regulation-driven. These properties are representative of end-of-pipe abatement technologies in general and representative of CCS specifically^51,52^. Some of the insights gained for FGD may therefore be transferrable to the case of CCS, though many uncertainties regarding the development of CCS should be considered, including technology choice, storage options, technology scaling, system integration, economic and financial viability, politics and regulations and public acceptance^11^.

In direct comparison to FGD, CCS faces a unique set of challenges. First, transport and storage of CO_2_ are different from transport and storage of the by-products of FGD (mainly gypsum that can be used in the cement industry). Second, the alternatives to CCS technology are more abundant than the alternatives to FGD upon its introduction; whereas sulfur reduction through other means was unattractive, CCS faces stiff competition from a range of low carbon energy technologies that can substitute coal power wholesale. Third, the investment and operational costs imposed by CCS on coal power plant owners are likely to be more significant than for FGD.

In terms of policy and politics, the analogy between FGD and CCS is useful insofar governments are not aiming to phase out coal altogether. Current leaders on the CCS policy front include Norway, the UK, the US, China, Canada and Japan^53^. Whereas Germany was a leader in the introduction of FGD, it is unlikely to drive the adoption of CCS, because of a commitment to phasing out rather than cleaning up coal^54^. Government support for CCS has been limited to a small number of rich countries that heavily rely on fossil fuels, and appears almost independent of political consensus over the importance of climate change^55^; for example, the US decided to withdraw from the Paris Agreement but also provides a tax credit for CCS^56^.

Finally, our findings contribute to the debate in the literature regarding the temporal dynamics of energy transitions^18,19,21^. Our analysis confirms that diffusion can occur relatively rapidly when it involves just the conversion and supply layer of the energy system^19^. Besides, the diffusion rates of FGD in Germany, Japan, South Korea and China confirm that rapid transitions are characterised by a low degree of technological complexity, significant benefits and strong policy frameworks^18^. FGD could diffuse faster than coal power itself because it is less complex, reduces SO_2_ very strongly and was guided by strict regulatory requirements. At the same time, larger markets and early adopters generally feature slower diffusion^18^, which is confirmed by the patterns in the US (large, early and slow), but contradicted by the patterns for Germany (large, early, but fast).

Further work should focus on the diffusion of FGD in other industries than coal power and possible analogies with CCS for such industries. It should also consider the role of partial substitutes to FGD, which include coal washing, fuel switching and cutting operational hours. It can be difficult to draw the boundary in this regard since, for example, technology switching has very different costs and benefits compared to installing an end-of-pipe technology for existent capacity. It would also be useful to compare FGD with other end-of-pipe technologies, such as air pollution control at waste incineration plants, wastewater treatment technologies and other industry-specific pollution control technologies. A comparison of various end-of-pipe technologies could provide a more fine-grained insight into the likely diffusion patterns of CCS.

## Methods

### Aims and framework

This study aimed to identify key patterns in the diffusion of FGD. Our dependent variables describe the level of diffusion of FGD and are, first, the absolute capacity of FGD (MWe) and second, the market share in coal capacity. The study includes both absolute capacity and market share (i.e., a normalised indicator) to account for the fact that market growth allows wider diffusion of technologies^5^. The focus is on capacity but the framework includes unit numbers to reveal the contribution of unit size growth to overall capacity growth^4^.

We looked at the contribution of unit numbers (-) and unit size (MWe) to the absolute capacity of sulfur control and analysed relative contributions through decomposition of the data. For both unit numbers and unit size, we considered four types of capacity: conventional combustion with new build FGD, conventional combustion with retrofit FGD, conventional combustion without FGD and advanced combustion methods that do not require FGD.

To assess the rates of diffusion, times of adoption and saturation levels, we conducted logistic curve regressions and calculated the following indicators: ∆*t*, which describes the time to go from 10 to 90% of the saturation level, the time of adoption *t*_0_ and the saturation level K.

### Coal power data

We used a global database of coal power plants with FGD adoption data to analyse patterns of FGD diffusion from 1970 till 2010, collated by the International Energy Agency (IEA). The database covers the year of commissioning, year of decommissioning, electric capacity (MWe), year of introduction of FGD and current FGD status. The analysis is based on the measurement of units, i.e., coal power units, of which there might be several in a single plant. Coal power plants that are either FBC or IGCC were identified based on the keywords ‘fluidised’ and ‘gasification’ in the boiler information.

Minor data cleaning operations—such as correction of unusual formats for various entries—are listed in Supplementary Note 1. The main concern with the IEA database is an underestimation of FGD installations in recent years. The data were crosschecked with, to the authors’ best knowledge, independent references and corrected when deemed necessary. A summary of country-level comparisons with literature sources is provided in Supplementary Table S8. This comparison led to minor adjustments to the data for Japan and South Korea.

For China, we found that the figures for 2006–2010 are underestimated in the IEA database, most likely because of the unprecedented rate at which power stations were built. We replaced the data with power sector figures from the Chinese Electricity Council (CEC)^57^ and a database on Chinese coal power units with FGD from the Chinese Ministry of Ecology and Environment (MEE)^58^. These data correspond closely with other CEC documents cited in the literature^59^. A full description of the data sources and assumptions is provided in the Supplementary Note 2.

### Rates of diffusion

The materiality boundary represents 1% of global primary energy use^24^. We calculated the equivalent in coal capacity based on global quantities of coal designated for the power sector as reported by the International Energy Agency (IEA)^60^. We calculated the equivalent of materiality in coal power capacity for 1971 (the earliest year in the reference for primary energy) and 2010 to estimate the range.

The logistic regressions are based on the standard logistic diffusion equation as a function of time (*t*) with *K* the saturation level, *b* the steepness of the curve and *t*_0_ the curve midpoint (with *t*_0_ = 0 for the year 1970).

$$f\left( t \right) = \frac{K}{{1 + e^{ - b(t - t_0)}}}$$

Diffusion patterns may be summarised and compared using metrics that reflect the main properties of the diffusion curve, some of which are not intuitive (for example, it is hard to interpret b as a measure of the rate of diffusion). Arguably the best indicator is ∆*t*, which is calculated as follows, and which refers to the number of years required to move from 10 to 90% of the saturation level^20^.

$${\mathrm{{\Delta}}}_t = \frac{1}{b} * {\mathrm{ln}}(81)$$

The number 81 reflects the choice of boundaries (10 and 90%). For other intervals, the number would be different (e.g., for 20–80% it would be 64).

The irregular stepwise pattern of diffusion in some countries leads to either an under or overestimation over the saturation level. In regressions for market share, the saturation level was constrained to 1; in regressions for absolute capacity, the saturation level was constrained to total coal power capacity in 2010.

### Decomposition analysis

The contribution of each type of unit—retrofit FGD, new build FGD and FBC/IGCC—can be directly inferred from the respective installed capacities. The following equation describes the total change in capacity with sulfur control (*S*) and is simply the sum of changes in retrofit (*R*), new build (*N*) and alternative combustion (*A*) capacity.

$$\left( {{\mathrm{{\Delta}}}_S} \right)_{tot} = \left( {{\mathrm{{\Delta}}}_S} \right)_R \,+\, \left( {{\mathrm{{\Delta}}}_S} \right)_N \,+\, \left( {{\mathrm{{\Delta}}}_S} \right)_A$$

The contribution of unit numbers (*#*) and average installed capacity (*P*) can be broken down into the following components.

$$\left( {{\mathrm{{\Delta}}}_S} \right)_R \,\,=\, S^{t,R} - S^{0,R} = \left( {{\mathrm{{\Delta}}}_S} \right)_{{\mathrm{\# }},R} \,+\, \left( {{\mathrm{{\Delta}}}_S} \right)_{P,R}$$

$$\left( {{\mathrm{{\Delta}}}_S} \right)_N \,\,=\, S^{t,N} - S^{0,N} = \left( {{\mathrm{{\Delta}}}_S} \right)_{{\mathrm{\# }},N} \,+ \, \left( {{\mathrm{{\Delta}}}_S} \right)_{P,N}$$

$$\left( {{\mathrm{{\Delta}}}_S} \right)_A \,\,=\, S^{t,A} - S^{0,A} = \left( {{\mathrm{{\Delta}}}_S} \right)_{\# ,A} \,+\, \left( {{\mathrm{{\Delta}}}_S} \right)_{P,A}$$

However, the contribution of changes in numbers and the average size of units of a certain type towards the installed capacity of the same type must be analysed through decomposition analysis; the capacity of, for example, retrofit FGD is the product of number and size and a simple calculation of the contribution of each factor therefore has multiple solutions^61^. To circumvent this issue, we used the Logarithmic Mean Divisia Index (LMDI) decomposition method^62^ with the individual contributions being calculated as follows:

$$\left( {{\mathrm{{\Delta}}}_S} \right)_{{\mathrm{\# }},R} \,\,=\, \frac{{S^{t,R} - S^{0,R}}}{{\ln S^{t,R} - \ln S^{0,R}}}\ln \frac{{R^{{\mathrm{\# }},t}}}{{R^{{\mathrm{\# }},0}}}$$

$$\left( {{\mathrm{{\Delta}}}_S} \right)_{P,R} \,\,=\, \frac{{S^{t,R} - S^{0,R}}}{{\ln S^{t,R} - \ln S^{0,R}}}\ln \frac{{R^{P,t}}}{{R^{P,0}}}$$

$$\left( {{\mathrm{{\Delta}}}_S} \right)_{\# ,N} \,\,=\, \frac{{S^{t,R} - S^{0,R}}}{{\ln S^{t,R} - \ln S^{0,R}}}\ln \frac{{N^{\# ,t}}}{{N^{\# ,0}}}$$

$$\left( {{\mathrm{{\Delta}}}_S} \right)_{P,N} \,\,=\, \frac{{S^{t,R} - S^{0,R}}}{{\ln S^{t,R} - \ln S^{0,R}}}\ln \frac{{N^{P,t}}}{{N^{P,0}}}$$

$$\left( {{\mathrm{{\Delta}}}_S} \right)_{\# ,A} \,\,=\, \frac{{S^{t,R} - S^{0,R}}}{{\ln S^{t,R} - \ln S^{0,R}}}\ln \frac{{A^{\# ,t}}}{{A^{\# ,0}}}$$

$$\left( {{\mathrm{{\Delta}}}_S} \right)_{P,A} \,\,=\, \frac{{S^{t,R} - S^{0,R}}}{{\ln S^{t,R} - \ln S^{0,R}}}\ln \frac{{A^{P,t}}}{{A^{P,0}}}$$

### Properties of the diffusion curves

For a reliable comparison of duration, extent and time of adoption, only regression curves that met the following criteria were included: the diffusion in 2010 should be at least 60% of the saturation level (extent) calculated in the same regression and the *R*^2^ should be at least 0.95, consistent with the criteria in the previous literature^34^. We also excluded countries that still had no FGD by 2005. Both the regression for capacity and market share needed to meet the criteria for a country to be included in either analysis. We plotted the log of extent in absolute capacity because of the large variability in market sizes.

### Modelling scenarios comparison

CCS diffusion scenarios were taken from two scenario databases. The first is the SR15 database of scenarios used to inform the IPCC’s Special Report on 1.5 °C of Global Warming^3,47^. This database provides data on a range of 1.5 °C and 2 °C average global warming scenarios, for coal CCS, gas CCS and bioenergy CCS. Data are available from three models (AIM/CGE, REMIND and IMAGE) for 1.5 °C scenarios, and from two models (REMIND and AIM/CGE) for 2 °C scenarios. The second scenario database is the AMPERE database hosted by IIASA^48,49^, which provides scenario data on coal CCS capacity, by region, for eight integrated assessment models. We used AMPERE3-450, a scenario to explore possible pathways for stabilising atmospheric CO_2_ at 450 ppm (approximately representing a 2 °C scenario). For normalised comparisons, we used each model’s global GDP projection and global GDP data^63^ for normalising historic diffusion of FGD. Both historical and future global GDP was based on market exchange rates. We also compared FGD with the historical diffusion of other power generation technologies, using the same sources for global GDP, and a variety of sources for technology diffusion data^34,64,65^.

### Limitations

Measuring capacity does not capture the possible reduction in operational hours of coal power units without FGD to reduce overall SO_2_ emissions. However, a focus on actual electric output instead of installed capacity is not necessarily preferable since energy production depends on a host of factors, only one of which is limiting sulfur emissions—such an analysis may thus not lead to very useful conclusions regarding sulfur control. A focus on capacity has advantages because FGD capacity is installed for no other reason than to reduce SO_2_ emissions.

Our analysis did not include sulfur control through fuel switching and reduction of operational hours of high sulfur plants. Fuel switching can be a workable alternative to FGD, though it cannot achieve the same removal rates of SO_2_ emissions^66^, and many plants may have done this, particularly in contexts where the capital investment required for FGD installations proved a barrier. Fuel switching to low sulfur coal can have significant benefits though switching to gas leads to much greater reductions. The purchasing of low sulfur coal can be costly but does not require the upfront investment that is needed for FGD.

It should be kept in mind that FGD adoption does not always mean actual operating of FGD systems. A recent study found that reported sharp reductions in Chinese SO_2_ emissions could not be corroborated with satellite data, suggesting misreporting^67^. Whilst the causes could be many, there is a possibility that the necessary upfront technology investment was made but that plant owners subsequently proved unwilling to accept the operational costs of continuous flue gas cleaning (which includes additional fuel requirements due to efficiency losses and waste treatment costs). Our analysis was limited to capacity installation, not operation.

### Reporting summary

Further information on research design is available in the Nature Research Reporting Summary linked to this article.

## Supplementary information

Supplementary Information

Reporting Summary

## Data Availability

Relevant aggregated data are available from the corresponding author upon reasonable request. The IEA coal power database is no longer publicly available; all other data used for this study are publicly available (see references). Source data are provided with this paper.

## Source data

Source Data
