# Supplementary material for: Diffusion of flue gas desulfurization reveals barriers and opportunities for carbon capture and storage
Source: Nat Commun. 2020 Aug 27;11:4298. doi: 10.1038/s41467-020-18107-2 (PMC7453001; doi:10.1038/s41467-020-18107-2)
Supplement: Supplementary file 1 — Supplementary Information [file 41467_2020_18107_MOESM1_ESM.pdf]

Supplementary Information

Diffusion of flue gas desulfurization reveals barriers and opportunities for carbon capture and storage

Van Ewijk et al.

Supplementary Figures

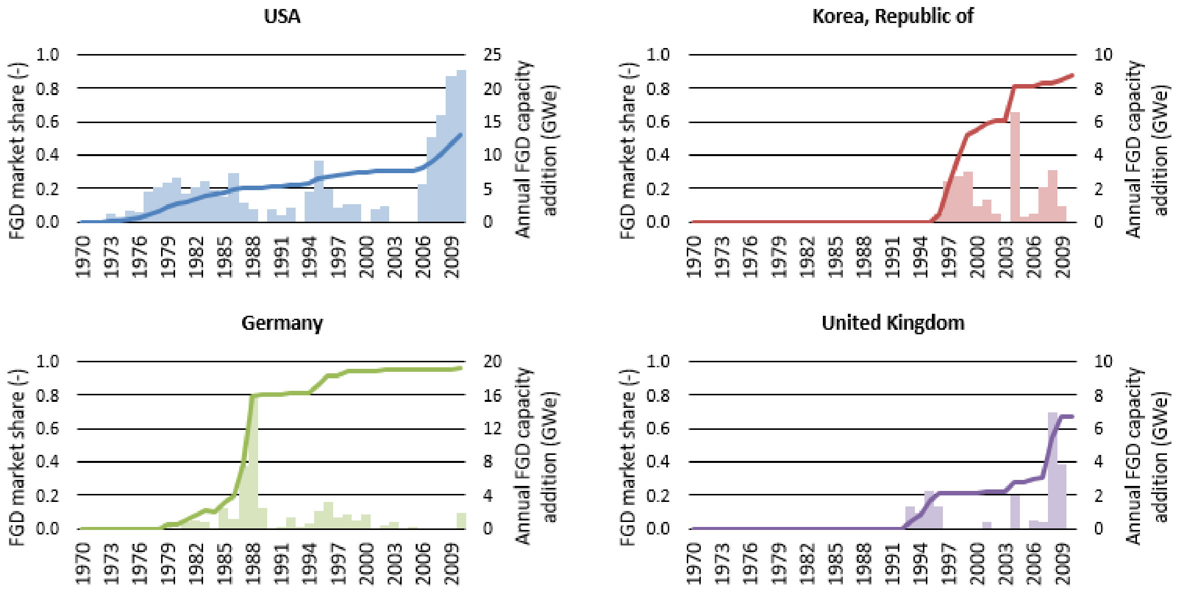

Supplementary Figure 1. Rapid pulses of diffusion of FGD (retrofit and new build) in selected countries. The primary axis shows the market share and the secondary axis the annual growth capacity growth rates. For each country 2 or 3 pulses can be observed. The exact number of pulses depends on whether the more modest pulses are included (for example, the 2nd pulse in the US is more modest than the first and third).

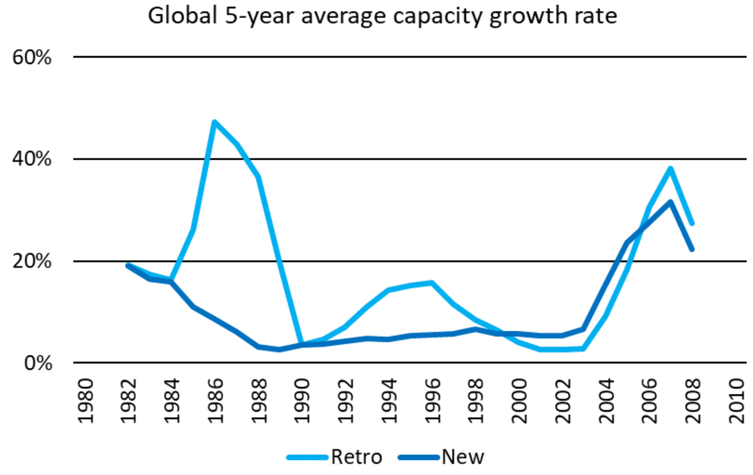

Supplementary Figure 2. Five-year average growth rates for retrofit and new build FGD at the global level. Growth in retrofit FGD capacity tends to outpace growth in new build FGD capacity.

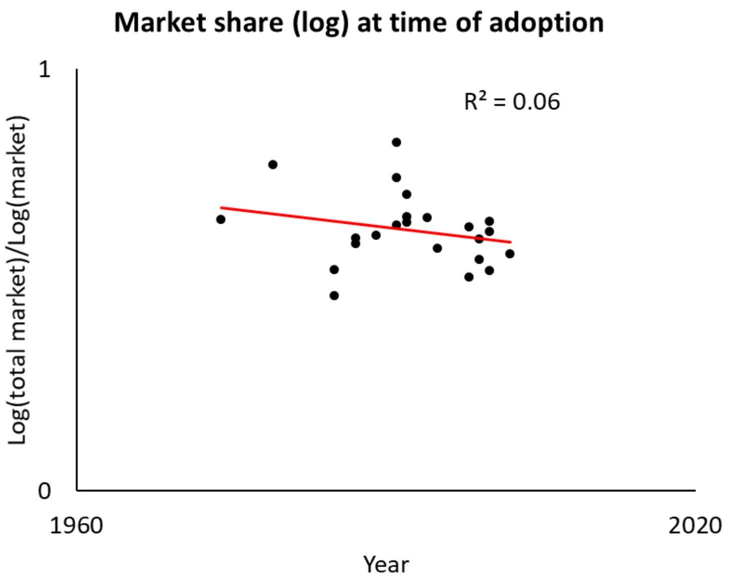

Supplementary Figure 3. Global market share at time of adoption. Market share is calculated as the log of national coal capacity (MWe) divided by the log of global coal capacity (MWe) in the year of adoption. The same exclusion criteria were used as for the analysis of duration, extent, and time of adoption.

Supplementary Tables

Supplementary Table 1. Regression statistics and indicators for market shares and installed capacity in selected countries.

	Parameters	China	USA	Japan	South Korea	Germany	Poland	United Kingdom	Global
Market share	t0	36.6	24.3	12.4	29.0	17.0	27.1	38.1	40.4
	K*	0.91	0.49	0.90	0.83	0.92	0.47	1.00	1.00
	b	0.98	0.10	0.31	0.54	0.79	0.51	0.18	0.10
	R squared	1.00	0.92	0.99	0.98	0.99	0.99	0.91	0.93
	Delta t (years)	4	44	14	8	6	9	24	44
	Extent (%)	91%	49%	90%	83%	92%	47%	100%	100%
Capacity	t0	37.4	27.7	27.0	32.6	18.5	28.0	38.1	40.2
	K**	609	181	42	27	46	15	30	1,635
	b	0.95	0.10	0.18	0.34	0.39	0.43	0.19	0.17
	R squared	1.00	0.92	0.99	0.99	0.98	0.99	0.92	0.93
	Delta t (years)	5	45	25	13	11	10	23	26
	Extent (GWe)	609	181	42	27	46	15	30	1,635

*Constrained to 1. **Constrained to installed national coal capacity in 2010.

Supplementary Table 2. Regression statistics and indicators for rapid pulses of diffusion in Germany and South Korea.

Parameters	South Korea	Germany
Time period	1970-2003	1970-1994
t0	27.5	16.8
K	0.59	0.82
b	1.34	1.25
R squared	1.00	0.98
Delta t (years)	3	4
Extent (%)	59%	82%

Supplementary Table 3. Duration for two dependent variables and two types of FGD for selected countries. Only values for regressions with R^2 higher than 0.95 were included.

	China	USA	Japan	South Korea	Germany	Poland	United Kingdom	Global
Delta t capacity	5	-	25	13	11	10	-	-
Retrofit	4	-	17	-	2	10	-	-
New build	5	13	22	15	23	-	-	-

Supplementary Table 4. Comparison of historical FGD diffusion with other power sector technologies based on maximum global historical deployment rates.

	Maximum deployment (GW/decade/$T GDP)	
**Technology**	**Up to 2010**	**Up to 2017 or 2018**
Coal FGD	11.1	-
Nuclear	5.0	5.0
Coal and oil	8.3	9.7
Natural gas	8.8	8.8
Hydro	3.1	4.0
Wind	2.5	5.4
Solar PV	0.6	5.7

Supplementary Table 5. Maximum deployment rates in 1.5 °C scenarios. Green cells are below the maximum historical rate of FGD, whereas red cells exceed the historically observed record for FGD. Note that the REMIND column includes scenarios from the linked REMIND-Magpie model.

		Coal CCS			Gas CCS			BECCS		
Model	FGD history	AIM/CGE	IMAGE	REMIND	AIM/CGE	IMAGE	REMIND	AIM/CGE	IMAGE	REMIND
Absolute growth (GW/decade)	731	1,023	278	63	1,521	854	164	2,465	1,015	360
Normalised growth (GW/decade/ $T of GDP)	11	6.2	0.8	0.5	8.6	4.4	0.7	13.3	6.2	2.3

Supplementary Table 6. Maximum deployment rates in 2 °C scenarios. Green cells are below the maximum historical rate of FGD, whereas red cells exceed the historically observed record for FGD. Note that the REMIND column includes scenarios from the linked REMIND-Magpie model.

		Coal CCS		Gas CCS		BECCS	
Model	FGD history	AIM/CGE	REMIND	AIM/CGE	REMIND	AIM/CGE	REMIND
Absolute growth (GW/decade)	731	1,354	73	1,422	160	2,198	144
Normalised growth (GW/decade/ $T of GDP)	11	8.5	0.6	6.2	1.2	10.7	0.4

Supplementary Table 7. Maximum deployment rates of coal CCS in the AMPERE 450 scenario. Green cells are below the maximum historical rate of FGD, whereas red cells exceed the historically observed record for FGD.

Model	FGD history	MERGE-ETL	MESSAGE	IMAGE	REMIND	DNE21	WITCH	IMACLIM	POLES
Absolute growth (GW/decade)	731	1081	80	585	3	822	208	4060	788
Normalised growth (GW/decade/ $T of GDP)	11	8.2	0.6	2.4	0.0	8.1	2.1	36.6	7.8

Supplementary Table 8. Reliability check of data for selected countries.

Country	References	Notes
China	[1–3]	These references reveal large inconsistencies for recent years. The diffusion data was therefore updated, which is further explained under "Update of Chinese data".
US	[4]	There is some inconsistency between this reference and our data. However, the EPA data is for "net summer dependable capacity", which at least partially explains the discrepancies.
Japan	[5]	We cross-checked the data for a few major units that were recorded as not having FGD by 2010, which led to minor adjustments in the commissioning and FGD introduction years.
South Korea	[6]	Because of an uncommon increase in units without sulfur control, we conducted cross-checks at the plant level, and corrected the FGD status for a small number or large units commissioned in the 2000s.
Germany	N/A	Saturation of FGD adoption at approximately 100% of the total coal power market precludes underestimation.
Poland	[7]	This reference is consistent with the database. However, only a single data point was available for comparison, which is FGD capacity in 2001.
United Kingdom	[8]	This reference is consistent with the database.

Supplementary Notes

Supplementary Note 1: IEA database cleaning

Various entries in the database exhibited inconsistencies, such as the format of the data, and were therefore manually corrected. These corrections are of the following types.

- For one unit, we corrected the capacity and year of commissioning, which had been accidentally swapped.
- For five units, we chose the last year of the range indicated for the introduction of FGD. For example, “2007/-08” was changed to 2008.
- For one unit with advanced combustion, we removed a mistakenly entered year of introduction for FGD.
- For one unit, we manually entered the FGD introduction year based on an online article.
- For two units, we changed nonsensical decommissioning dates into the dates found for units commissioned in the same year in the same country.

These units make up a very small proportion of the in total 7,363 units in the database.

In some cases, the year of FGD installation preceded the year of power plant commissioning, most often by just one or two years. In such cases, we adjusted the year of FGD installation to the year the plant was commissioned. Sometimes FGD was indicated as “operational” but no introduction year was stated (2.8% of all units in the database, representing 2.6% of capacity); an attempt to address this by assuming new built FGD (i.e., inserting an FGD introduction year equal to the commission date of the unit) led to incorrect early instances of FGD and was therefore abandoned.

Supplementary Note 2: Data for China

The coal capacity and FGD share in China in the IEA database are significantly lower than in the literature [1]. We therefore consulted a variety of other data sources and replaced the IEA figures with the following references. First, the total coal capacity in China was taken from data published by the Chinese Electricity Council (CEC) and made available in English through China Energy Portal [3]. We cross-checked the figures with the original reports available directly from the CEC [2]. We approximated coal power capacity in 2006-2008 based on the thermal power capacity for these years and the average fraction of coal in thermal power capacity in 2009-2010.

Second, the new build and retrofit FGD capacity was taken from a database by the Chinese Ministry of Ecology and Environment (MEE) [9]. The data covers commissioning dates and years of introduction of FGD for units that were in operation by the end of 2013; it excludes units that were decommissioned before the end of 2013. The data is representative for earlier years since plants fitted with FGD are unlikely to have been closed down in the 2006-2010. Besides, the data corresponds closely to the fraction of (both retrofit and new build) FGD in the Chinese coal power market claimed by the CEC and cited elsewhere [1].

As an additional crosscheck, we compare total global coal power capacity with figures from the Global Coal Plant Tracker by Global Energy Monitor, which provides up-to-date information on coal power capacity worldwide [10]. After correction of the Chinese data, the total global power capacity in 2010 in our estimate differs by 1.7% from the estimate in the Tracker.

Supplementary References

1. Schreifels, J. J., Fu, Y. & Wilson, E. J. Sulfur dioxide control in China: Evolving policies of the 10th and 11th Five-year Plans and lessons for the future. *Energy Policy* **2030**, 1–23 (2011).
2. China Electricity Council. Annual data (in Chinese). (2020).
3. China Energy Portal. Detailed Electricity Statistics. (2020). Available at: https://chinaenergyportal.org/en/2010-detailed-electricity-statistics/.
4. US EPA. National Electric Energy Data System (NEEDS) v.5.15. (2018).
5. Kyushu Electric Power Co Inc. *Start of power generation of Matsuura Power Station Unit 2*. (2019).
6. Ki Suh Park. FGD Technologies for Korean Coal Firing Power Station. in *International Conference on Transboundary Air Pollution in North-East Asia* (2008).
7. Galos, K. A., Smakowski, T. S. & Szlugaj, J. Flue-gas desulphurisation products from Polish coal-fired power-plants. *Appl. Energy* **75**, 257–265 (2003).
8. Markusson, N. The politics of FGD deployment in the UK (1980s-2009) Final case study report as part of Work Package 2 of the UKERC project : „ CCS – Releasing the Potential ?‟. (2012).
9. Ministry of Ecology and Environment. National coal-fired unit desulfurization facilities (in Chinese). 3–181 (2014). Available at: http://www.mee.gov.cn/gkml/hbb/bgg/201407/W020140711581927228220.pdf.
10. Global Energy Monitor. Global coal power. *Carbon Brief* (2018). Available at: https://www.carbonbrief.org/mapped-worlds-coal-power-plants. (Accessed: 2nd February 2019)
