# Supplementary material for: Diffusion of flue gas desulfurization reveals barriers and opportunities for carbon capture and storage
Source: Nat Commun. 2020 Aug 27;11:4298. doi: 10.1038/s41467-020-18107-2 (PMC7453001; doi:10.1038/s41467-020-18107-2)
Supplement: Supplementary file 2 — Reporting Summary [file 41467_2020_18107_MOESM2_ESM.pdf]

nature research

Corresponding author(s): Stijn van Ewijk

Last updated by author(s): Jul 3, 2020

Reporting Summary

Nature Research wishes to improve the reproducibility of the work that we publish. This form provides structure for consistency and transparency in reporting. For further information on Nature Research policies, see Authors & Referees and the Editorial Policy Checklist .

Statistics

For all statistical analyses, confirm that the following items are present in the figure legend, table legend, main text, or Methods section.

n/a	Confirmed	
☐	☒	The exact sample size (*n*) for each experimental group/condition, given as a discrete number and unit of measurement
☒	☐	A statement on whether measurements were taken from distinct samples or whether the same sample was measured repeatedly
☒	☐	The statistical test(s) used AND whether they are one- or two-sided<br>*Only common tests should be described solely by name; describe more complex techniques in the Methods section.*
☒	☐	A description of all covariates tested
☒	☐	A description of any assumptions or corrections, such as tests of normality and adjustment for multiple comparisons
☐	☒	A full description of the statistical parameters including central tendency (e.g. means) or other basic estimates (e.g. regression coefficient) AND variation (e.g. standard deviation) or associated estimates of uncertainty (e.g. confidence intervals)
☒	☐	For null hypothesis testing, the test statistic (e.g. *F*, *t*, *r*) with confidence intervals, effect sizes, degrees of freedom and *P* value noted<br>*Give P values as exact values whenever suitable.*
☒	☐	For Bayesian analysis, information on the choice of priors and Markov chain Monte Carlo settings
☒	☐	For hierarchical and complex designs, identification of the appropriate level for tests and full reporting of outcomes
☐	☒	Estimates of effect sizes (e.g. Cohen's *d*, Pearson's *r*), indicating how they were calculated

Our web collection on statistics for biologists contains articles on many of the points above.

Software and code

Policy information about availability of computer code

Data collection	No software was used for data collection. The data was obtained as MS Excel (IEA) or PDF (Ministry of Ecology and Environment) file.
Data analysis	The data was analysed in MS Excel (Office 365 2016). Relevant files are available from SVE upon request.

For manuscripts utilizing custom algorithms or software that are central to the research but not yet described in published literature, software must be made available to editors/reviewers. We strongly encourage code deposition in a community repository (e.g. GitHub). See the Nature Research guidelines for submitting code & software for further information.

Data

Policy information about availability of data

All manuscripts must include a data availability statement. This statement should provide the following information, where applicable:
- Accession codes, unique identifiers, or web links for publicly available datasets
- A list of figures that have associated raw data
- A description of any restrictions on data availability

Relevant aggregated data are available from the corresponding author upon reasonable request. The IEA coal power database is no longer publicly available; all other data used for this study is publicly available (see references). Source data for the article figures are provided as a Source Data file.

Field-specific reporting

Please select the one below that is the best fit for your research. If you are not sure, read the appropriate sections before making your selection.

☐ Life sciences ☐ Behavioural & social sciences ☒ Ecological, evolutionary & environmental sciences

Ecological, evolutionary & environmental sciences study design

All studies must disclose on these points even when the disclosure is negative.

Study description	Analysis of patterns of diffusion of FGD in the coal power sector, in absolute capacity and market share, using logistic curve regression, linear regression, and decomposition analysis.
Research sample	Coal power units in power plants in 77 countries in 1970-2010
Sampling strategy	The initial analysis focused on the 7 largest national coal power sectors with significant diffusion of FGD, and the global coal power sector. The further analysis included the 50 largest coal power sectors in the database. Results from logistic regressions exclude countries for which the following criteria could not be met: at least 60% diffusion of the saturation level in 2010 and an R2 of at least 0.95. This left 23 countries in the sample.
Data collection	The data was obtained as MS Excel (IEA) or PDF (Ministry of Ecology and Environment) file.
Timing and spatial scale	Global, 77 countries, 1970-2010
Data exclusions	Corrections were made to a small number of database entries (see Supplementary Notes 1-2). Data for China was taken from a separate publicly available database (see manuscript and Supplementary Note 3).
Reproducibility	We did not conduct experiments.
Randomization	We did not conduct experiments.
Blinding	We did not conduct experiments.

Did the study involve field work? ☐ Yes ☒ No

Reporting for specific materials, systems and methods

We require information from authors about some types of materials, experimental systems and methods used in many studies. Here, indicate whether each material, system or method listed is relevant to your study. If you are not sure if a list item applies to your research, read the appropriate section before selecting a response.

Materials & experimental systems

n/a	Involved in the study
☒	☐ Antibodies
☒	☐ Eukaryotic cell lines
☒	☐ Palaeontology
☒	☐ Animals and other organisms
☒	☐ Human research participants
☒	☐ Clinical data

Methods

n/a	Involved in the study
☒	☐ ChIP-seq
☒	☐ Flow cytometry
☒	☐ MRI-based neuroimaging
